# Reflection-mode virtual histology using photoacoustic remote sensing microscopy

**DOI:** 10.1038/s41598-020-76155-6

**Published:** 2020-11-05

**Authors:** Kevan Bell, Saad Abbasi, Deepak Dinakaran, Muba Taher, Gilbert Bigras, Frank K. H. van Landeghem, John R. Mackey, Parsin Haji Reza

**Affiliations:** 1 Department of Systems Design Engineering, PhotoMedicine Labs, University of Waterloo, E7-6416 200 University Avenue West, Waterloo, ON N2L 3G1 Canada; 2 Department of Systems Design Engineering, illumiSonics, Inc., University of Waterloo, Waterloo, ON N2L 3G1 Canada; 3 Department of Oncology, University of Alberta, Edmonton, AB T6G 1Z2 Canada; 4 Division of Dermatology, Department of Medicine, University of Alberta, Edmonton, AB T6G 2V1 Canada; 5 Department of Laboratory Medicine and Pathology, University of Alberta, Edmonton, AB T6G 2V1 Canada

**Keywords:** Surgical oncology, Microscopy, Imaging and sensing, Molecular imaging, Microscopy

## Abstract

Histological visualizations are critical to clinical disease management and are fundamental to biological understanding. However, current approaches that rely on bright-field microscopy require extensive tissue preparation prior to imaging. These processes are both labor intensive and contribute to creating significant delays in clinical feedback for treatment decisions that can extend to 2–3 weeks for standard paraffin-embedded tissue preparation and interpretation, especially if ancillary testing is needed. Here, we present the first comprehensive study on the broad application of a novel label-free reflection-mode imaging modality known as photoacoustic remote sensing (PARS) for visualizing salient subcellular structures from various common histopathological tissue preparations and for use in unprocessed freshly resected tissues. The PARS modality permits non-contact visualizations of intrinsic endogenous optical absorption contrast to be extracted from thick and opaque biological targets with optical resolution. The technique was examined both as a rapid assessment tool that is capable of managing large samples (> 1 cm^2^) in under 10 min, and as a high contrast imaging modality capable of extracting specific biological contrast to simulate conventional histological stains such as hematoxylin and eosin (H&E). The capabilities of the proposed method are demonstrated in a variety of human tissue preparations including formalin-fixed paraffin-embedded tissue blocks and unstained slides sectioned from these blocks, including normal and neoplastic human brain, and breast epithelium involved with breast cancer. Similarly, PARS images of human skin prepared by frozen section clearly demonstrated basal cell carcinoma and normal human skin tissue. Finally, we imaged unprocessed murine kidney and achieved histologically relevant subcellular morphology in fresh tissue. This represents a vital step towards an effective real-time clinical microscope that overcomes the limitations of standard histopathologic tissue preparations and enables real-time pathology assessment.

## Introduction

**Figure 1:**
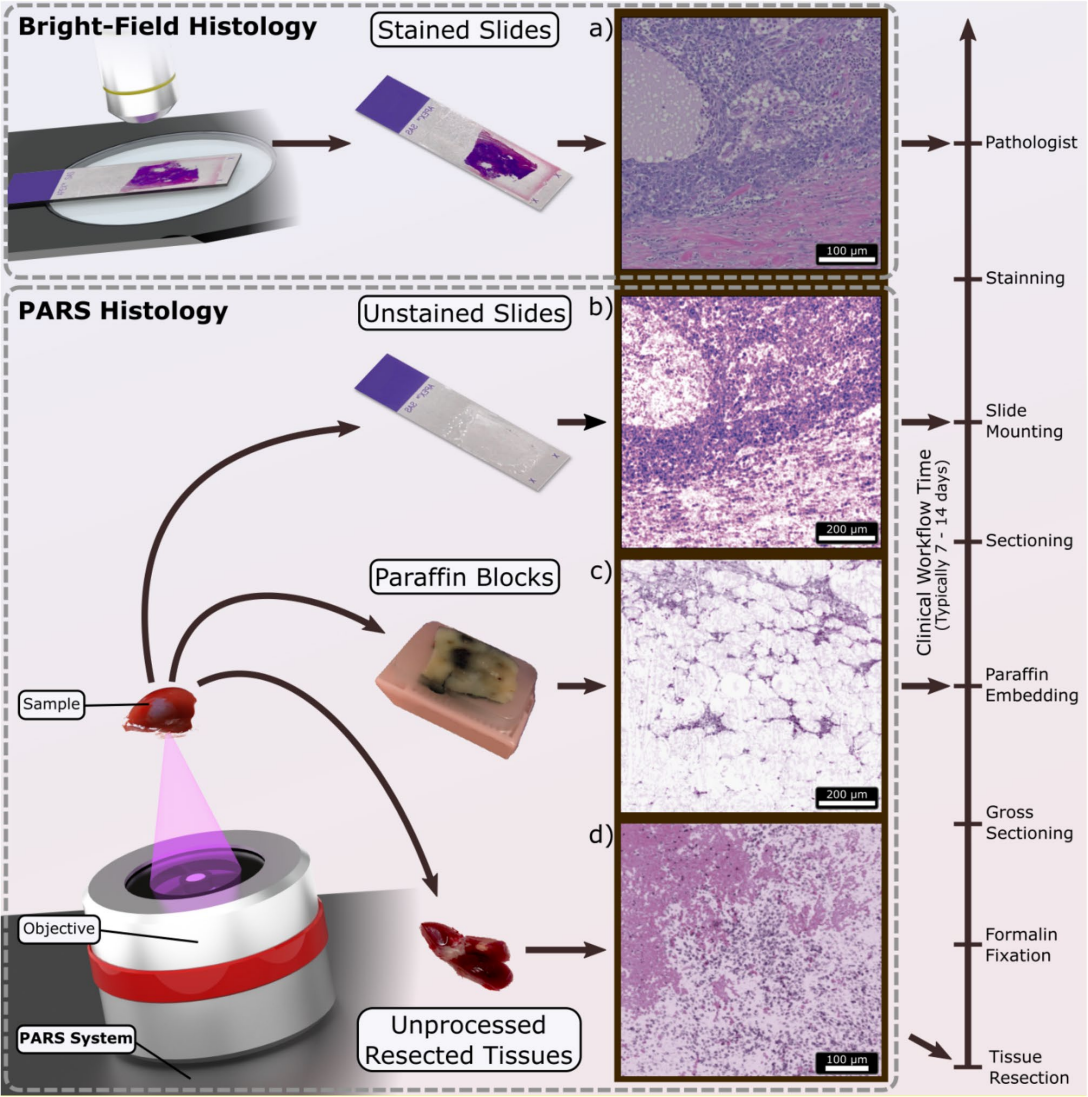
Overview of PARS histologic imaging workflow as compared to conventional light microscopy. (**a**) Conventional imaging of H&E-stained slides is performed on a bright-field microscope where the Hematoxylin (purple hues) and Eosin (red hues) stains block light from a white source. PARS may image (**b**) unstained FFPE slide preparations, (**c**) unstained FFPE blocks and (**d**) unprocessed tissues by taking advantage of the intrinsic optical absorption provided by the cell nuclei (DNA) and the surrounding cytoplasm (cytochrome). We image each intermediate step along the FFPE process in this paper using a single system configuration to show the versatility of PARS. No other reported technique has reported all of these capabilities in a single modality.

Visualizing tissue pathology plays a central role in surgical oncology, cancer screening, drug development, and biological research. The standard histopathology workflow produces thin sections of tissue that are typically stained with dyes such as hematoxylin and eosin (H&E). These dyes then highlight specific sub-cellular contrast. For example, hematoxylin highlights acidic regions such as nuclei in purple and eosin shows basic regions such as cytoplasmic filaments in muscle cells, intracellular membranes, and extracellular fibres in pink^1^. The preparation of histology slides, however, requires a potentially laborious multi-step process^2^. Tissue resected from biopsies or surgeries is typically fixed in formalin for up to 24 h, then dissected. Representative samples are oriented, dehydrated (in which tissue water is replaced by alcohol, then xylene) and infiltrated with and embedded in paraffin wax to create a tissue block. The tissue blocks are sectioned with a microtome into approximately 4–5 micron sections then placed on glass slides. The paraffin is removed from the tissue by a graded series of solvents, the tissue is then rehydrated, and finally stained with H&E. The slide is then commonly interpreted by a pathologist using a transmission light microscope. Figure 1 illustrates the multiple steps of this process. This complex workflow can commonly require two days to one week within a clinical setting before a diagnostic report can be issued. The clinical turnaround time for complex specimens, such as radical cancer resections, may be greater than ten days for some cases^3^. Intraoperative tools such as frozen section analysis are commonly employed to guide surgical management and achieve negative resection margins, but this technique rapidly freezes the specimens and can introduce artifacts into the tissue morphology that could hinder clinical interpretation^4^. An ideal imaging method would produce H&E-like diagnostic quality images directly on unprocessed freshly resected tissue. This would save valuable time during biopsy assessment, permit more rapid intraoperative assessment of surgical margins, guide total resection of the tumor and reduce re-operation rates.

A microscope which can disrupt the standard histopathology workflow and provide H&E-like contrast directly within the resection site could radically change the clinical pathology paradigm. However, such a device would need to meet several key requirements: (I) The device must be capable of emulating common existing methods. Pathologists are accustomed to assessing stained tissues and such a device must be capable of producing comparable visualizations of cellular structures with appropriate resolution and chromophore-specific contrast. Such visualizations may also be leveraged by existing AI recognition systems which have been previously trained on conventional histology preparations for use in cancer detection and surgical guidance. (II) The device must be capable of reflection-mode imaging. It is typically challenging for transmission-mode microscopes to visualize morphology on thick specimens such as freshly resected tissue or directly within the resection site. (III) The device must be capable of label-free visualization of intrinsic endogenous contrast. Exogenous dyes can be toxic and may require additional safety measures in clinical or surgical environments. (IV) The microscope must not require contact with the target in order to reduce the risk of infection and permit a rapid disinfection process between cases. (V) The device must be capable of real-time feedback. Real-time imaging would provide immediate feedback during surgeries and confirm suitability of tissue acquired in biopsy procedures. (VI) The device must be capable of 3-dimentional imaging or optical sectioning. Optical sectioning provides a means to visualize multiple layers of diseased tissue without the need for physical sectioning. (VII) Finally, it would be desirable if the microscope were able to image specimens at each intermediate step (as shown in Fig. 1) during the standard histopathological process. This would enable parallel integration into existing workflows at hospitals and encourage adoption. These capabilities, when combined, would result in a microscope that is suitable for intraoperative environments and would facilitate diagnostic quality H&E-like contrasts in fresh tissue specimens or directly within resection sites.

A variety of techniques have been developed to provide an alternative to standard histopathology. These methods have yet to be widely adopted as they do not fully address the requirements described above. Techniques such as fluorescence microscopy^5–8^, structured light microscopy^9,10^, light-sheet microscopy (LSM)^11^ and microscopy with ultraviolet surface excitation (MUSE)^12,13^ have demonstrated promising results in providing H&E-like contrast on tissue mounted on microscope slides or freshly excised tissue. However, these methods cannot image unstained tissue and require the application of fluorescence dyes to the sample, adding time, expense, and the potential for occupational exposure to these chemicals. LSM has reported rapid volumetric imaging of unfixed tissue but requires additional processing steps such as optical clearing of the samples in addition to fluorescence dyes^11^. Optical coherence tomography (OCT) has been used for virtual H&E imaging with the method reporting cellular scale resolutions^14,15^. However, as OCT uses optical scattering to provide contrast it is not capable of easily differentiating chromophores due to lack of specificity. Since H&E staining is chromophore specific, OCT images do not typically resemble H&E slides requiring pathologists to be retrained to interpret OCT visualizations^16,17^. Stimulated Raman scattering (SRS) modalities have provided label-free optical imaging^18^. However, these devices have primarily been shown in a transmission-mode architecture, limiting samples to thin sections. Transmission-mode SRS microscopes have demonstrated H&E-like contrast in thin and unfixed tissue specimens without the use of exogenous dyes^19,20^. However, thick tissue was imaged by tightly squeezing the sample between coverslips which is unsuitable for imaging large specimens or directly imaging a resection bed^21^. In addition, the squeezing procedure may place considerable pressure on the sample, potentially damaging cellular morphology, interfering with accurate margin assessment due to distortion, and hindering the analysis of the sample with standard histopathological techniques.

Photoacoustic (PA) imaging techniques such as optical-resolution photoacoustic microscopy (OR-PAM) have demonstrated exceptional visualizations of nuclear and cytoplasm morphology^22–24^. PA imaging takes advantage of the endogenous optical absorption contrast present within tissue along with various contrast agents which may be tagged to desirable regions of interest. These techniques have found utility in accessing deeper targets ($\gg$ 1 mm) by taking advantage of lower acoustic scattering within biological tissues and have demonstrated their own potential benefits for cancer diagnosis^25–27^. To detect absorption contrast, traditional PA devices employ ultrasonic transducers (similar to ultrasound imaging systems) which are in contact with the target. The requirement for contact with the acoustic transducer poses significantly higher risks and logistical challenges in maintaining surgical field sterility. The transducers are typically bulky devices which make their application in space-constrained resection sites difficult. Moreover, most PA methods for histology-like imaging have been demonstrated in transmission-mode which makes them unsuitable for unfixed tissue or in-situ imaging^22,23^. PA imaging devices have previously used ring-shaped transducers for high resolution reflection-mode approaches. However, such devices require the tissue and parts of the apparatus to be submerged in water for effective acoustic coupling making them unfeasible for in-situ imaging^28^. These limitations, therefore, pose significant challenges for clinical or intraoperative applications.

Photoacoustic Remote Sensing (PARS) is an emerging non-contact photoacoustic imaging technique^29,30^. PARS circumvents the limitations of conventional PA techniques by replacing the acoustic transducer with a continuous-wave detection laser^31,32^. This laser provides an all-optical design which allows for reflection-mode non-contact label-free imaging. This is in contrast to some all-optical photoacoustic techniques which may employ surface-coupled acousto-optic transducers such as Fabry–Perot etalons^33^. These capabilities, as highlighted earlier, lead to a device suitable for intraoperative and clinical environments. Previously, PARS has demonstrated efficacy in recovering histopathological structures from within various preparations of human tissues in formalin-fixed paraffin embedded (FFPE) thin sections and blocks and frozen sections^34,35^. However, much of these previous efforts were conducted across various iterations of PARS devices and therefore do not necessarily provide a strong foundation for comparing the capabilities of a given PARS microscope across such preparations. As well, previous efforts revolving around fresh unprocessed samples have not yet provided convincing histopathological recovery. Here, we aim to provide a more comprehensive comparison of current PARS devices and their performance across various human tissue preparations including unstained frozen sections and unstained paraffin embedded samples (both slides and blocks), as well as freshly resected unstained murine tissues. Where possible these images are qualitatively compared to adjacent sections subject to the standard histopathological process.

## Results

**Figure 2:**
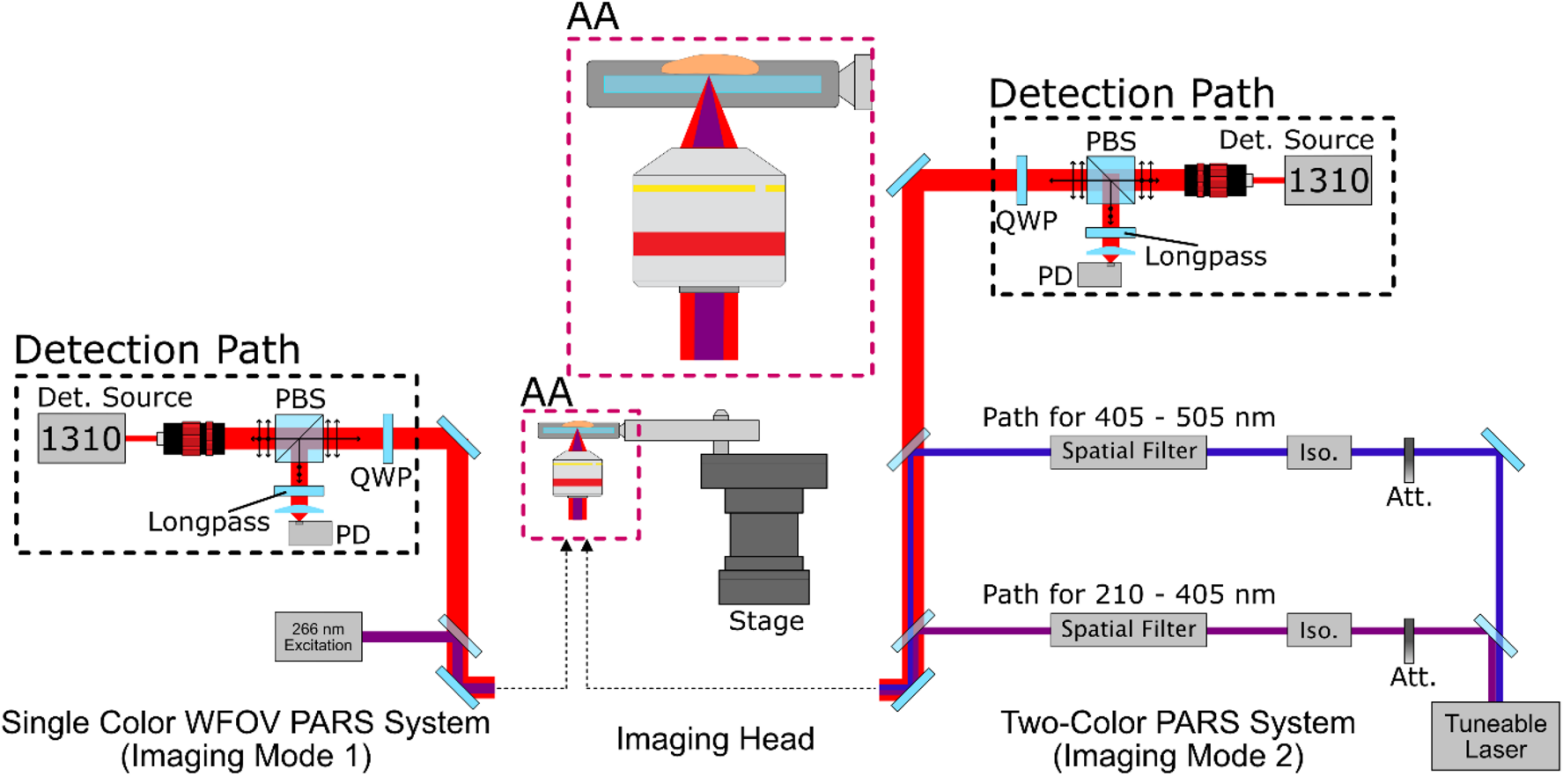
A simple system diagram showing the two excitation pathways. Component labels are defined as photodiode (PD), quarter wave-plate (QWP), Attentuator (Att), Isolator (Iso.).

In this study a primary goal was to replicate information provided by conventional H&E staining. Towards this end, we targeted the ultraviolet absorption peak of DNA (~ 260 nm) to extract nuclear contrast and the blue light (420 nm) absorption of cytochromes to extract cytoplasm contrast. Details regarding wavelength selection are included in Supplement Information 1 and 2. Imaging was conducted on two PARS imaging platforms (Fig. 2): (I) A single-color system which employs a single 266 nm excitation laser for targeting DNA contrast which operates at a repetition rate of 50 kHz. This system provided both rapid grossing capabilities (> 2 cm^2^) and near-diffraction-limited lateral resolution (~ 425 nm). To highlights system contrast, visualizations from this system are shown in grayscale with lighter regions representing large photoacoustic interactions (targeted optical absorption contrast) and darker regions representing low photoacoustic interaction (little to no targeted optical absorption contrast). (II) A two-color system^36^ which employs a tuneable excitation source configured to provide both 250 nm (for DNA) and 420 nm (for cytochromes) which operates at a repetition rate of 1 kHz. This lower repetition rate makes larger grossing scans less practical as compared to the faster single-color device, however, the tuneable nature of the excitation allows for more precise control and exploration of ideal absorption wavelengths. False-coloring is applied to these two-color acquisitions attempting to emulate the look of corresponding images of H&E preparations. Both devices are implemented using a 1310 nm detection laser providing optimal imaging penetration depth within the optically scattering samples^37^. Images are formed by mechanical scanning of the sample using a pair of scanning stages. Further details regarding system layout and design are provided in “Methods” and Supplementary Information 3. System sensitivity characteristics, including pulse energies used, were explored for the various tissues featured in this study, these results are included in Supplementary Information 5.

**Figure 3:**
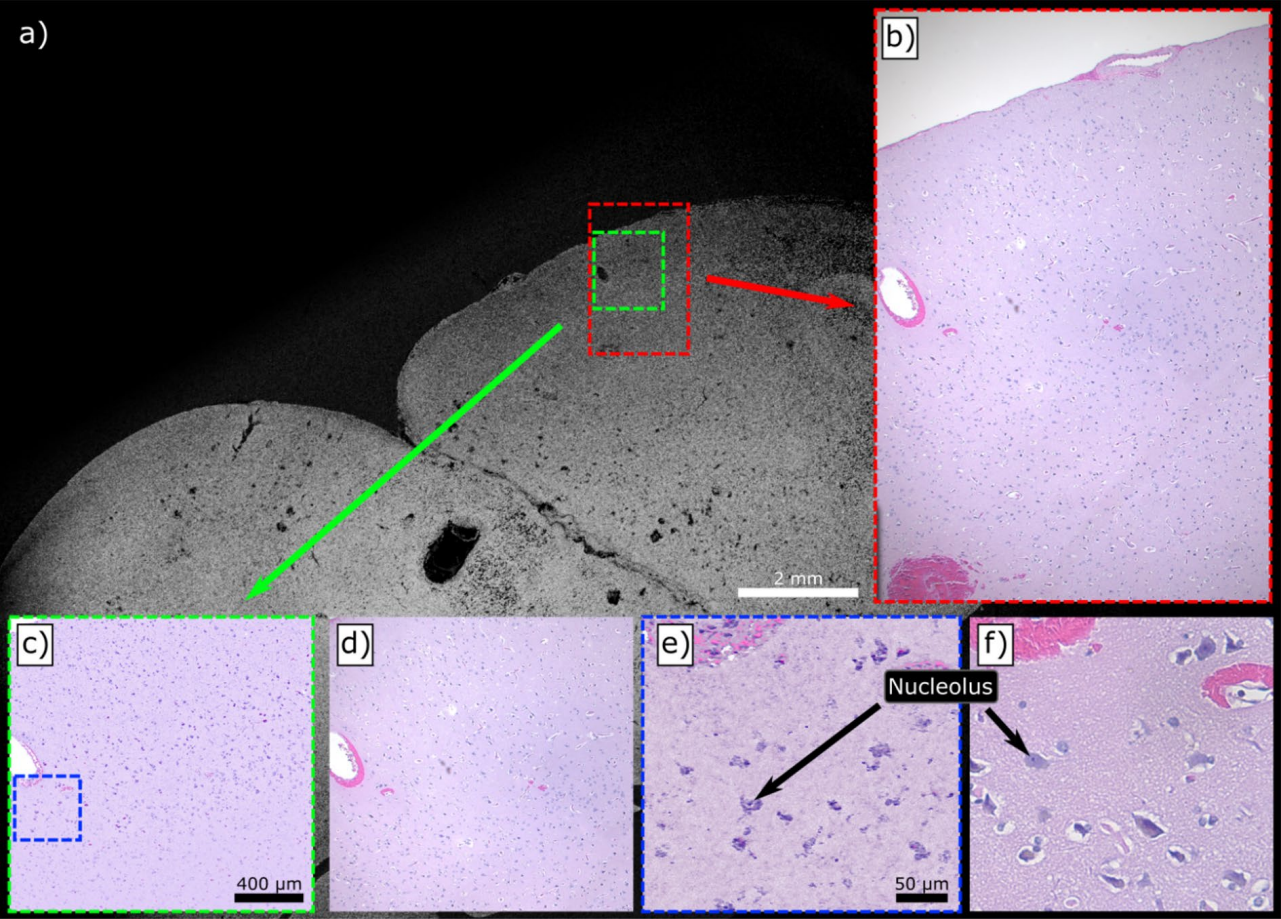
Several comparisons between PARS and conventional bright-field images of FFPE slides of human brain tissues. (**a**) A wide field of view (WFOV) scan using 266 nm excitation with (**b**) a matching wide field image of the adjacent slide which has been H&E stained. (**c**) A two-color (250 nm and 420 nm) PARS with a false-colour map applied to match (**d**) the adjacent H&E region. Finally (**e**) and (**f**) likewise show a two-color PARS and bright-field image respectively in higher detail.

The first tissue preparation method explored in this work is that of FFPE slides. Apart from the lack of any exogenous staining, this sample type is quite similar to the conventional H&E slides which would be viewed under bright field microscopes. They are flat and highly transparent without visible pigmentation. FFPE slide preparations of human brain tissues are presented in Fig. 3. Gross imaging of the entire specimen is provided by the single-color system (Fig. 3a) in a 21 mm × 13 mm acquisition. The adjacent slide underwent conventional H&E-staining for comparison, a large section of which is shown in Fig. 3b and its relative location on the PARS image is shown in red. Two-color acquisitions were then performed on smaller regions (Fig. 3c,e) and are presented with their adjacent H&E counterparts (Fig. 3d,f). Here, PARS demonstrates its ability to recover the sparse nuclear structure within this healthy human brain sample. At this scale, further diagnostic qualities are accessible such as internuclear spacing and nuclear volumes. These features may facilitate the differentiation of normal brain tissue, grey and white matter but also identification of specific cell types such as neurons, glial cells or endothelial cells. The 420 nm contrast (shown as pink) provided additional morphology information such as the location of blood vessels through high concentrations of erythrocytes and provided additional structural information within the internuclear regions.

**Figure 4:**
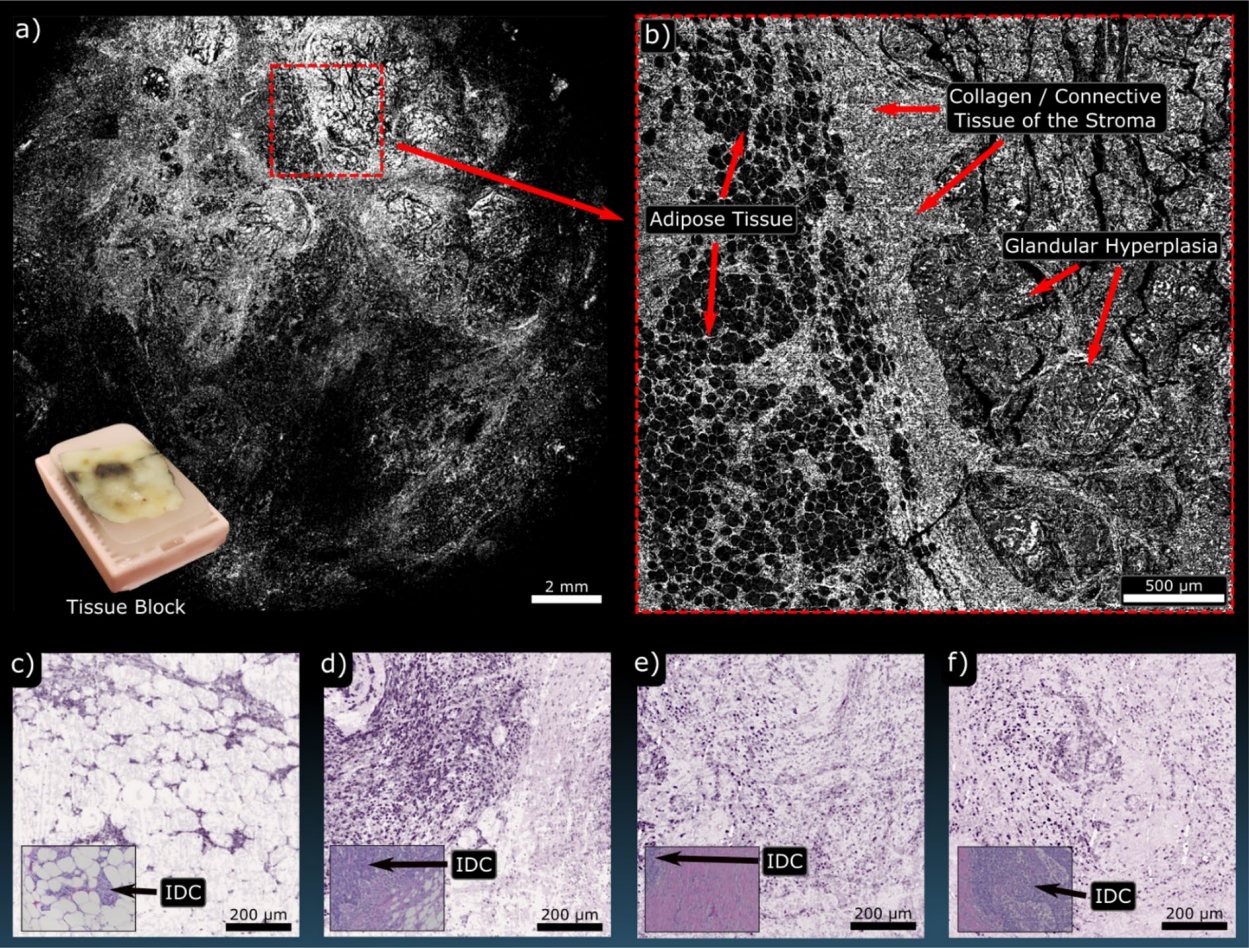
PARS imaging performed on FFPE tissue blocks of human breast. (**a**) Highlights a WFOV single-color 266 nm acquisition covering nearly the entire tissue block surface. Inset is an image of the tissue block which was imaged. (**b**) shows a higher resolution single-color 266 nm of the highlighted region in red. At these scales bulk tissue components can be identified such as adipose tissue and fibroglandular tissue. (**c**–**f**) shows several two-color acquisitions from FFPE tissue blocks of human breast with bright-field images of similar H&E-stained regions inset. These all highlight regions of invasive ductal carcinoma (IDC).

**Figure 5:**
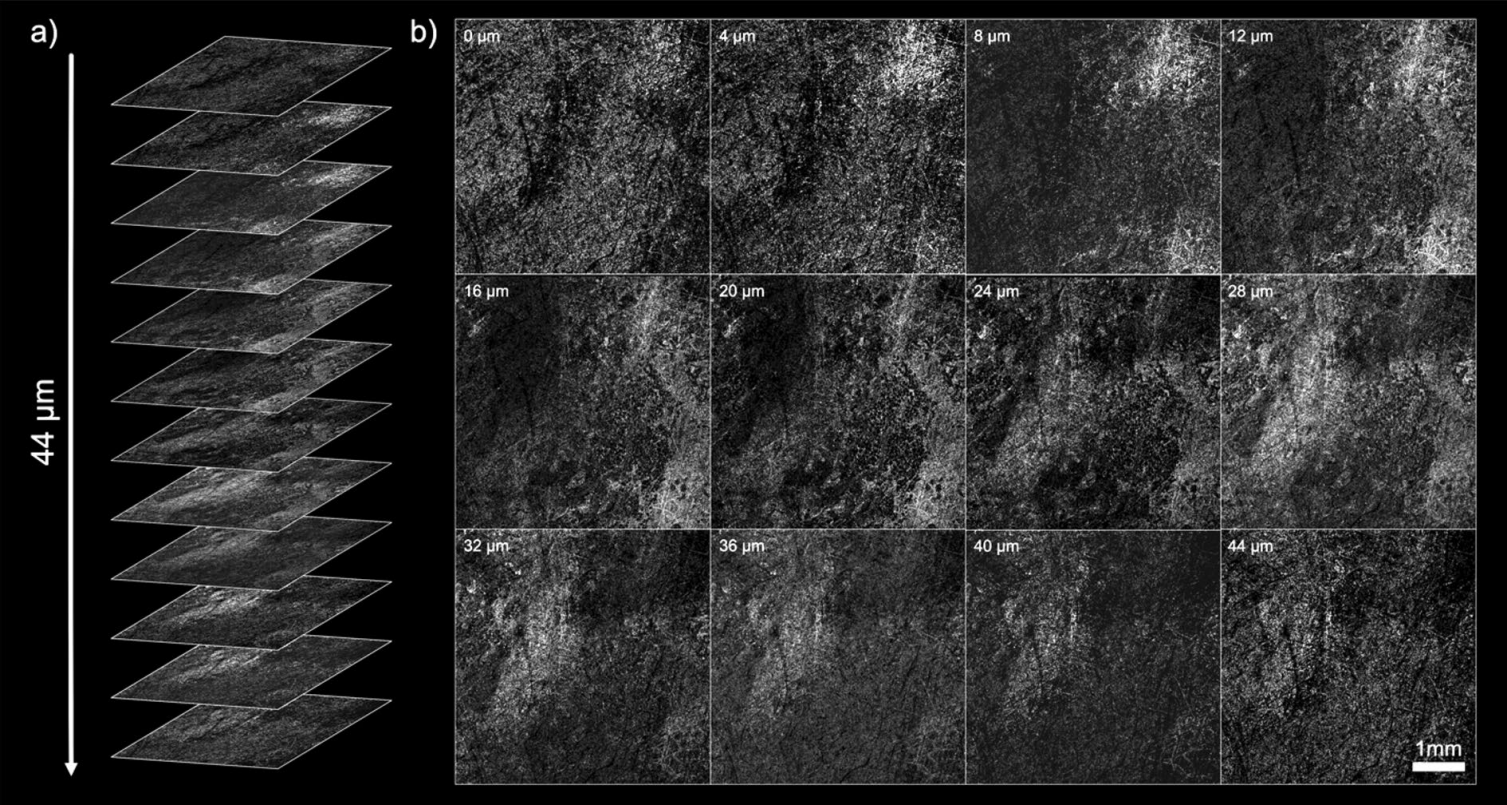
Several 2D sections from a 3D PARS scan of a FFPE human breast tissue block. (**a**) Shows the various slides which constitute the volume in a stack. (**b**) Shows several of the sections in greater detail.

PARS imaging of FFPE tissue blocks of human breast tissue with ductal carcinoma in situ (DCIS) followed. Tissue blocks from breast tissue resections are typically several millimeters thick and highly opaque, making transmission-mode modalities impractical. Blocks imaged here had adjacent sections removed by a microtome, leaving a flat surface for the PARS microscopes to image. Single-color acquisitions are shown in Fig. 4a which highlights a wide grossing scan (17 mm × 17 mm) of the block surface and Fig. 4b which highlights a higher-resolution scan (3 mm × 3 mm) of the region marked in red. At this resolution adipose tissue, stromal elements and regions of DCIS are clearly visible. Yet smaller scans (800 μm × 800 μm) were then performed at further locations on the block surface using the two-color PARS (Fig. 4c–f). For comparison, inset into these figures are bright-field H&E images of similar regions. The difference in contrast between the fat cells and the surrounding tissues becomes more apparent with the addition of the 420 nm hemeprotein contrast. The results also demonstrate that PARS can recover fine nuclear structure from within these FFPE blocks. This represents the first time that large sections of FFPE tissue blocks have been visualized by taking advantage of the intrinsic optical absorption contrast of DNA. Continuing with the same FFPE human breast tissue blocks, volumetric acquisitions were performed on the single-color system (Fig. 5). These were acquired by taking consecutive 2D scans (5 mm × 5 mm) at various respective depths within the sample. Unique bulk structures can be seen at each level demonstrating the optical sectioning capabilities of the PARS technique. As well, since each depth level is separated by around 4 µm, each represents morphology which would be recovered on a single FFPE slide. In this study, distinct regions were observed down to around 44 μm. As such, this technique may be useful in providing rapid virtual sectioning of the tissue block negating the need for multiple slides. When combined with the high imaging rate, these capabilities facilitate future development of a PARS-based rapid FFPE block grossing tool.

**Figure 6:**
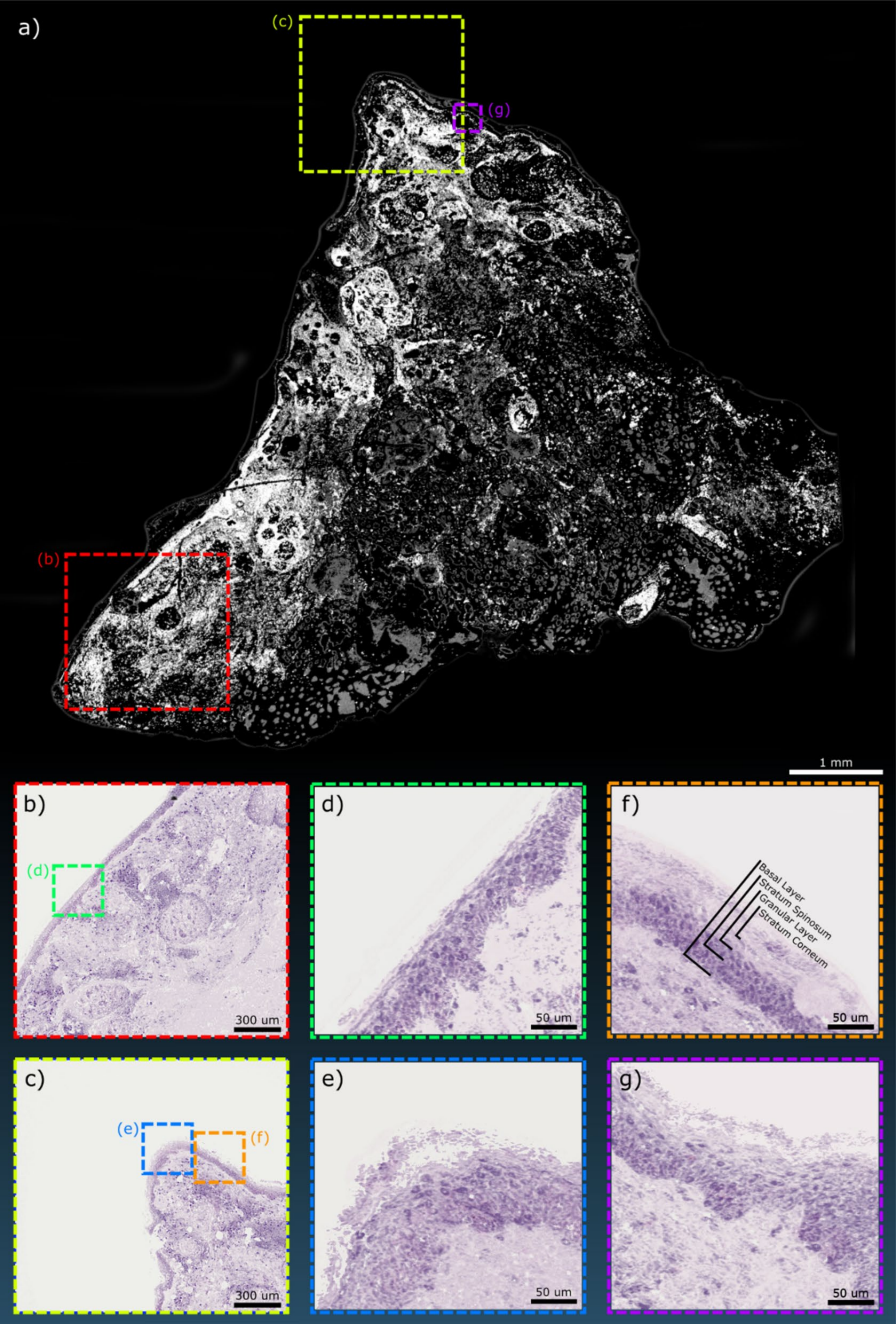
Several PARS images of a human skin sample mounted as a frozen section slide. (**a**) A WFOV PARS acquisition of the sample using the single color 266 nm system. The two-color PARS was then used over smaller field the views in (**b**,**c**) focusing on the outer tissue layers. Still smaller field of views are shown in (**d**,**g**) highlighting the details available within the epidermal layers. These layers are annotated in (**f**).

Frozen sections were then investigated. Normal human skin samples were grossed with the single-color system (10 mm × 10 mm), with such frames requiring approximately 3 min to acquire (Fig. 6a). Switching to the two-color PARS system, smaller scans were captured focusing on the epidermal layers (1.6 mm × 1.6 mm) (Fig. 6b,c) followed by yet smaller acquisitions (300 μm × 300 μm) (Fig. 6d–g) to further enhance the detail of the epidermal layers. From these higher resolution images, the sublayers of the epidermis begin to show including the outer stratum corneum followed by the granular layer, stratum spinosum and finally the basal layer below that (Fig. 6f). The dermo-epidermal junction is also clearly visible along with the dermal papillae (Fig. 6d).

**Figure 7:**
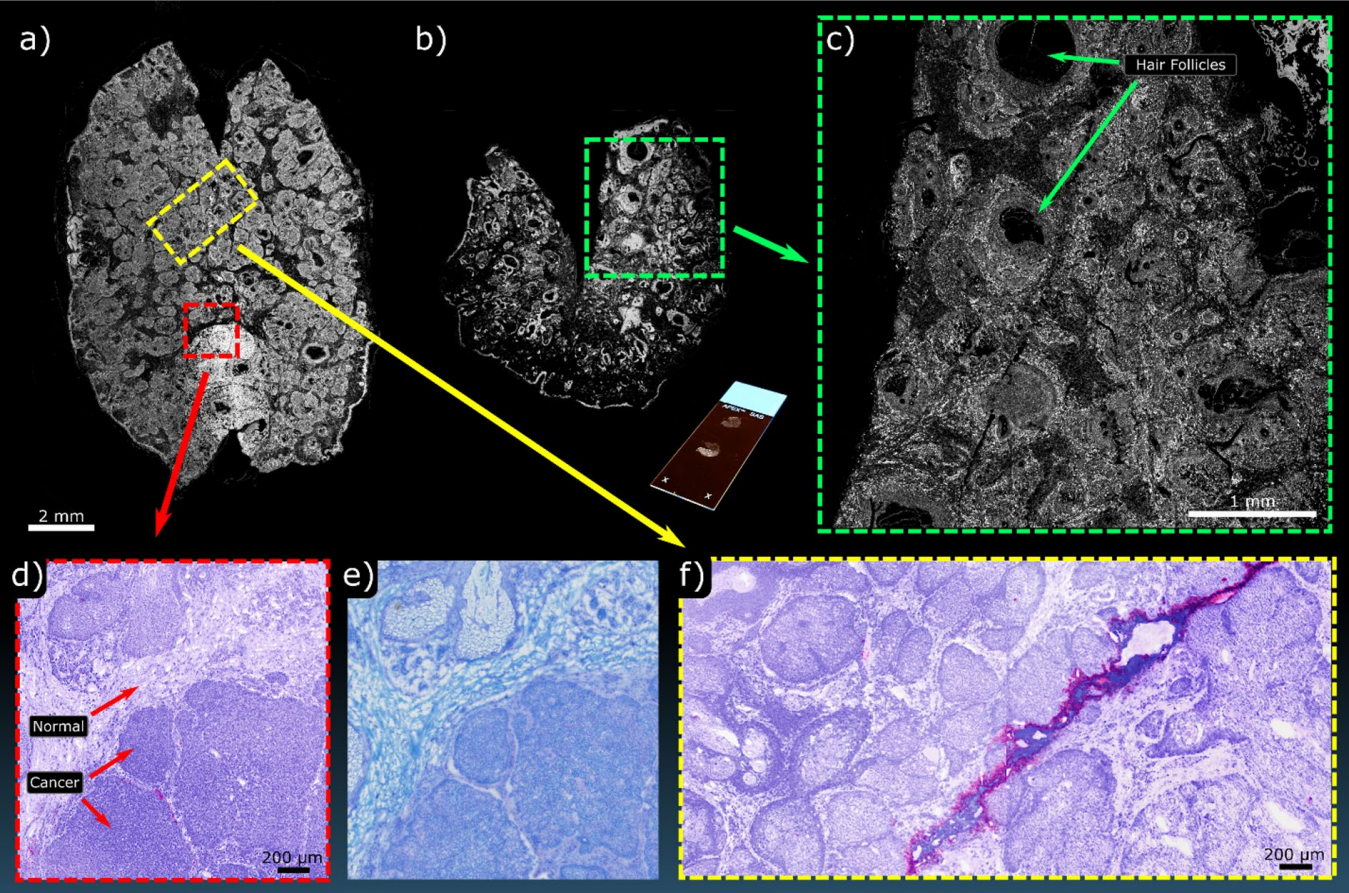
PARS imaging performed on frozen sections from a Mohs procedure. (**a**,**b**) Show WFOV single-color acquisitions of two separate entire frozen sections. Inset with (**b**) is an image of the unstained section mounted on a glass slide. (**c**) Shows a higher density scan of the highlighted region in (**b**). (**d**) shows a smaller FOV of the highlighted region in (**a**) captured with the two-color system along with (**e**) the corresponding section stained with toluidine blue captured on a standard bright-field microscope. (**f**) shows a region of subcutaneous healthy tissue captured on the two-color PARS which was likewise taken from the highlighted region in (**a**).

Next, frozen sections were acquired from a Mohs micrographic surgery procedure removing BCC from a human subject (Fig. 7). Standard frozen pathology was done and sections were stained using toluidine blue rather than H&E as this represented a common processing for BCC. Additional adjacent image slides from the same resection block were taken to provide unstained examples for PARS imaging. Here, two large grossing scans taken by the single-color PARS are presented which showed the entire frozen section (Fig. 7a,b). Figure 7c shows a smaller region taken from the highlighted area in Fig. 7b. This resolution level clearly resolves finer bulk structure and shows individual cell nuclei. Two-color acquisitions were then performed on the highlighted regions in Fig. 7a (Fig. 7d,f). These can then be compared with the adjacent sections stained in toluidine blue (Fig. 7e). Here, the overall morphological similarities between the bright-field image of the stained slide and the PARS image of the unstained slide is evident. Much of the finer detail in the zoomed-in regions can be seen, with morphological features of normal skin tissue versus cancerous tissue being appreciable (Fig. 7d). These results highlight the rapid diagnostic potential of the PARS technique working with frozen sections.

**Figure 8:**
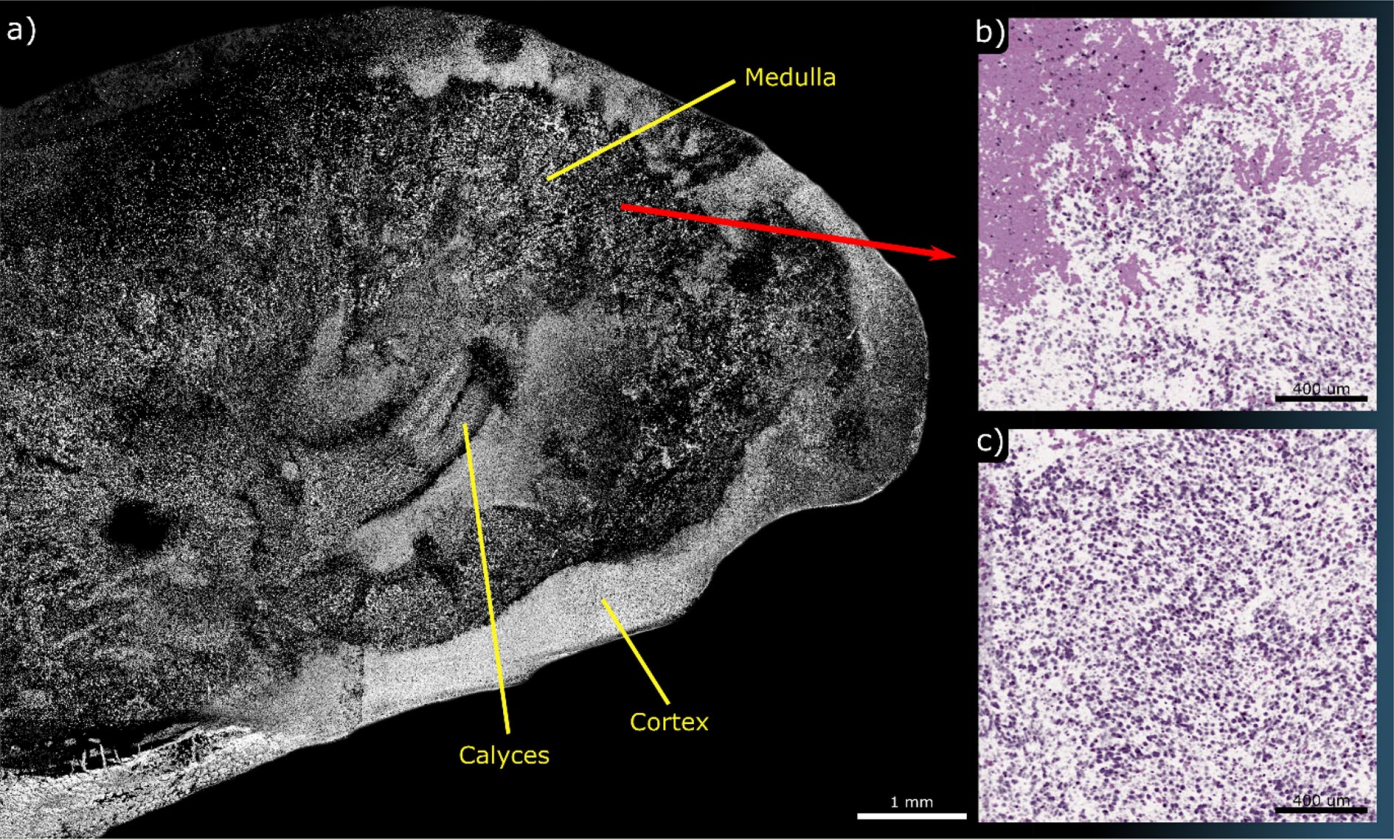
PARS imaging performed on unprocessed murine kidney. (**a**) shows a WFOV single-color acquisition of the organ which has been sectioned in half along the sagittal direction. Several bulk components are labeled. (**b**,**c**) shows smaller FOVs of the medulla region captured on the two-color system illustrating numerous tubule constituents of nephrons.

Fresh, unprocessed tissue was the final preparation studied in this work. Unprocessed murine kidneys were transported in room temperature PBS for transport and cut to produce sagittal sections which were then placed with the fresh cut against the UV viewing window of the system. A single-color grossing scan captured nearly the entire organ (Fig. 8a). Several bulk features can be identified including the Calyces, Medulla and Cortex. Several smaller regions were captured using the two-color system as shown in Fig. 8b,c. The smaller regions were taken around the medulla. Imaging required careful removal of excess fluids and blood by washing with fresh PBS, as the transport PBS produced measurable PARS signal without discernible morphology. This signal is assumed to be protein and other macromolecules which could provide non-zero signal under UV and blue light excitation. Despite these challenges, for the first time, PARS has demonstrated H&E-like visualizations of unprocessed tissue morphology. These experiments were performed with a large gap between the sample and the objective (> 7 mm) and without the use of any exogenous contrast agents. This represents a vital step towards PARS becoming an effective clinical tool as a method of rapid tissue assessment.

## Discussion

This work presented the efficacy of PARS microscopy in visualizing a wide variety of tissue preparations including human FFPE slides, FFPE tissue blocks, frozen pathology sections and unprocessed murine kidney. PARS was able to recover micron-scale tissue morphology solely by taking advantage of the intrinsic optical absorption properties of the tissue. The ability to image unstained fresh tissue directly may represent a practice changing technology for surgical management of cancer, which currently relies on lengthy processing methods. PARS microscopy functions both as a potential replacement for conventional bright-field processing techniques and as a rapid grossing tool capable of characterizing large regions of tissue. By presenting clinicians with rapid feedback within minutes, verified negative surgical margins may be achieved intra operatively more quickly and efficiently, reducing the need for additional surgeries. Furthermore, PARS maybe useful as a tool for virtual biopsy, to interrogate tissue prior to biopsy, as well as a rapid quality assurance step to determine if diagnostic quality tissue is obtained. Endoscopic configurations with PARS capability could reduce unnecessary biopsies and limit the load placed on pathology services, thereby improving patient outcomes and lowering healthcare costs. Finally, PARS interrogation of fresh tissue has the potential to identify biological features, such as lipid subtyping and cellular hydration, that are routinely lost in standard tissue preparation.

In this paper two separate excitation pathways were examined each with their own merits. The single-color PARS system performed rapid grossing of centimeter-scale tissue samples by using a 50 kHz 266 nm picosecond excitation source matched with a fast (300 mm/s) mechanical scanning stage. This hardware facilitates scan rates of around 1.6 s/mm^2^ at 4 µm steps allowing for large samples (> 1 cm^2^) to be visualized in under 8 min. Improved scanning methods, such as hybrid mechanical-optical scanning, adding galvanometer mirrors or polygon scanners, may allow a much faster interrogation rate with an order of magnitude improvement in imaging speed. If such a rate increase were accomplished entire tissue-block sized samples (~ 4 cm^2^) could be image within minutes with sampling at ~ 4 µm. The two-color tunable excitation source operated at a far slower 1 kHz pulse repetition rate but allowed for exploration of optimal histological wavelengths. Future systems for clinical deployment will use faster repetition rate non-tuneable sources at these optimal wavelengths. Such sources may also facilitate large multi-color grossing scans producing H&E-like acquisitions at rates more suitable for clinical use. Meanwhile, the tunable system remains a valuable tool for investigating additional contrast (lipids, melanin, histones, etc.). The current tunable system was relegated to smaller field of views to maintain pragmatic imaging times. Per wavelength area scan rates were substantially slower as compared to the single-color counterpart with performances of around 21 min/mm^2^ at 900 nm steps and around 178 min/mm^2^ at 300 nm steps. The two detection pathways proved complimentary, with the single-color version demonstrating the potential imaging speed of the method when used for gross assessment, and the two-color version demonstrating available contrast and H&E-like visualizations.

We emphasize that no sample-specific changes were made to the PARS microscopes between sample types, and no special preparation was applied to any of the samples prior to imaging. This is yet another pragmatic consideration that makes PARS unique among competing modalities such as MUSE^12^ or LSM^11^, in that samples can be directly imaged in their standard form. However, the system did perform differently on different tissue preparation types. The FFPE slides and tissue blocks provided lower signal intensities and higher background signal levels as compared to the frozen sections, possibly attributed to macromolecule degradation brought on by the process of formalin-fixation and embedding in paraffin, including denaturation and crosslinking of DNA. The more ubiquitous background signal seen in the samples is likely to be a result of the paraffin within and surrounding the tissues. Paraffin provides a non-zero signal at all three wavelengths used. However, paraffin’s lack of organized structure makes it easy to discern as background and separate from the tissue. In comparison, frozen section samples provided notably lower background and higher signal levels. These would be attributed to reduced DNA damage providing additional contrast, and lower signal provided by the background embedding material. However, the highest contrast was observed in unprocessed tissues which completely lacked background signal and provided similar contrast to that seen in the frozen sections. Moving forward, imaging of bulk tissue samples presents new challenges, including the uneven surface which may be left from the removal process. This requires either tracking of the sample surface (to be addressed in future works) or placing the sample against a window to provide a flat viewing surface suitable for standard PARS microscopy. Another challenge arising from unprocessed samples is the limited time available to image prior to tissue degradation after devitalization. For the purpose of this initial investigation, samples were imaged within 3 h of resection to minimize tissue degradation. By visualizing freshly resected tissue, PARS could provide rapid clinical feedback eliminating the need for further tissue processing.

Our studies demonstrate the range of applications to which PARS, a novel tissue imaging technology, can be applied to various tissue preparations. By virtue of the multiple pragmatic advantages inherent to this technology, including non-contact, label free, cellular level resolution with multi-wavelength contrast, and rapid reflection mode image acquisition, PARS represents a vital step towards an effective real-time clinical microscope that overcomes the limitations of standard histopathologic tissue preparations and enables real-time pathology assessment. With configurations optimized to individual clinical applications, the PARS platform technology has the potential to improve diagnostic and therapeutic workflows in a variety of clinical settings.

## Methods

This study examines human tissue in three different sample types: (I) unstained skin and brain tissue sections on glass slides (II) breast tissue fixed in formalin and embedded in paraffin (III) frozen sections of skin from Mohs surgery. Clinical collaborators at the Cross-Cancer Institute (Edmonton, Alberta, Canada) obtained samples from anonymous patient donors and removed all patient identification from the samples. The ethics committees waived the requirement for patient consent on the condition that samples were archival tissue no longer required for diagnostic purposes, and that no patient identifiers were provided to the researchers. The samples were obtained under a protocol approved by Research Ethics Board of Alberta (Protocol ID: HREBA.CC-18-0277) and University of Waterloo Health Research Ethics Committee (Humans: #40275 Photoacoustic Remote Sensing (PARS) Microscopy of Surgical Resection, Needle Biopsy, and Pathology Specimens). All human tissue experiments were performed in accordance with the relevant guidelines and regulations. In addition, freshly excised tissue from mice was obtained to demonstrate PARS’s performance in imaging unprocessed tissue (Photoacoustic Remote Sensing (PARS) Microscopy of Resected Rodent Tissues; Protocol ID: 41543). All murine tissue experiments were performed in accordance with the relevant guidelines and regulations. The preparation methods for all sample types are described below.

### FFPE sample preparation

To prepare FFPE blocks the tissue was submersed in formaldehyde for 48 h. The tissues were then dehydrated by repeatedly immersing the tissue in ethanol of increasing levels of concentration ending in a 100% concentration of ethanol. The tissues were thereafter cleared with xylene to remove ethanol and any residual fat tissue. This clearing permits molten paraffin wax (60° + C) to penetrate the tissue. This embeds the tissue in paraffin wax. As the paraffin cools to room temperature, the FFPE tissue blocks are mounted in a cassette and completed. Brain and breast tissue specimens are prepared using this process. To further prepare unstained thin tissue slices on glass slides, 5 µm ribbons are sectioned using a microtome and placed onto glass slides. The glass slides are then baked at 60 °C for 60 min to remove excess paraffin from the sections. A comparison stained section for brain tissue specimens was obtained by immediately cutting the next ribbon (within 10 μm), transferred to glass slides and then baked for 60 °C for 30 min. The specimens were then stained with H&E contrast dyes and covered with mounting media and a coverslip. Once the mounting media was dry, the slides were fully prepared. Note that the unstained sections were prepared without a cover slip as standard borosilicate glass cover slips are not transparent in 250 nm or 266 nm light.

### Frozen section sample preparation

The frozen sections for skin specimens with BCC were obtained via Mohs surgery. Tissue specimens of sizes up to 15 mm × 30 mm are embedded in an optimal cutting temperature compound and placed in a cryostat pre-cooled to − 20 to − 25 °C. The specimens are then frozen to the pre-cooled temperature for 1–10 min depending on tissue components and thickness (ex: dermal tissue required closer to 10 min, fatty tissue requires about 1 min). The frozen samples are then sectioned at a thickness of 5–10 microns and transferred to a warm (room temperature) microscopic slide. The slide is then air-dried and heat-fixed at 55 °C for 1 min. The sections are then stained with 1% toluidine blue aqueous solution, a common staining protocol for BCC. Once the staining has been performed, the slides are cover-slipped with mounting media.

### Unprocessed resected tissue sample preparation

To demonstrate imaging on unprocessed tissue, unprocessed murine kidney specimens were obtained with the aid of collaborators at the Central Animal Facility, University of Waterloo performing work under animal care approval (Photoacoustic Remote Sensing (PARS) Microscopy of Resected Rodent Tissues; Protocol ID: 41543). Kidneys were excised and immediately placed in PBS and imaging was conducted within 3 h of devitalization.

### System layout

The two PARS architectures used in this work are shown in detail in Supplementary Figure SI3. The single-color PARS system employs a 266 nm excitation laser with a 50 kHz repetition rate (WEDGE XF 266, Bright Solutions). This laser also outputs a 532 nm beam as a result of frequency doubling the primary 1064 nm wavelength. The 532 and 266 nm beams are separated using a CaF2 prism (PS862, Thorlabs Inc.). The 266 nm beam is then expanded using a variable beam expander (BE05-266, ThorLabs) and combined with the detection beam using a dichroic mirror (HBSY234, ThorLabs). The system utilizes a 1310 nm continuous-wave superluminescent diode detection laser (S5FC1018P, ThorLabs Inc.). This beam is polarized vertically and passed onto a polarizing beam splitter (CCM1-PBS254, ThorLabs Inc.). The polarizing beam splitter transmits most of the forward light towards the quarter waveplate where it is converted into circularly polarized light. The two beams are co-focused on the sample using a 0.3 NA reflective objective (LMM-15X-UVV, Thorlabs Inc.). Since the system is in an inverted configuration, the sample is placed on a UV-transparent optical window. The optical window is resting in a circular holder which is connected to the mechanical stages (XMS100-S, Newport Inc.) as shown in Fig. 2. The back-reflected light from the sample is then converted back to linearly polarized light using the quarter waveplate and directed towards the polarizing beam splitter. The polarizing beam splitter then reflects the majority of the back-reflected light towards the photodiode (PDB425C, Thorlabs Inc.). A long-pass filter with a 1000 nm cut-off (FELH1000, Thorlabs Inc.) blocks any 266 nm back-reflection letting only the 1310 nm beam reach the photodiode. This light is then focused onto the photodiode using an aspheric condenser lens (ACL25416U, Thorlabs Inc.).

The two-color PARS system utilizes a tunable source with a range of 210–2600 nm (NT242, Ekspla Inc.). The beam from this excitation laser is split into different optical pathways depending on the wavelength. This particular study primarily uses 250 nm and 420 nm wavelengths. The laser light is first split using a dichroic mirror (HBSY134, Thorlabs Inc.). Wavelengths less than 405 nm are focused into a pinhole using an achromatic doublet lens (ACA254-100-UV, Thorlabs Inc.) for spatial filtering. The filtered light is then collimated using a second doublet lens (ACA254-100-UV, Thorlabs Inc.) and passed through a beam expander (BE02-UVB, Thorlabs Inc.). The expanded light is combined with the detection beam using a dichroic mirror (HBSY234, Thorlabs Inc.). Wavelengths greater than 405 nm are split again using a dichroic mirror with a 505 nm cut-off (DMSP505, Thorlabs Inc.). Wavelengths between 405–505 nm are then spatially filtered, collimated and expanded in a similar process as the UV pathway. Wavelengths greater than 505 nm are spatially filtered by focusing the light into a single mode fiber. The light from the optical fiber is collimated and combined with the 405–505 nm path using a dichroic mirror (DMSP505, Thorlabs Inc.). The resulting optical path is combined with the detection beam using a subsequent dichroic mirror (DMLP1000, Thorlabs Inc.).

The tuneable PARS system uses a similar optical path for detection as the single-color PARS system. It also employs a 1310 nm superluminescent diode (SLD1018P, Thorlabs Inc.) which is directed onto the sample and back to the photodiode using the same combination of polarizing beam splitter and quarter waveplate. This light is combined with rest of the optical paths using dichroic mirrors. The combined light is then focused onto the sample using a 0.5 NA reflective objective (LMM-40X-UVV, Thorlabs Inc.). The back-reflected light from the sample is focused onto the photodiode (PDB425C, Thorlabs Inc.). A long pass filter (FELH1000, Thorlabs Inc.) ensures no excitation wavelengths influence the photodiode. The remaining 1310 nm light is then focused onto the photodiode using an aspheric condenser lens (ACL25416U, Thorlabs Inc.).

### False-color formation

Tissue stained with histological dyes exhibits rich colours and contrast. Stains colour different tissue components in patterns that a pathologist is trained to recognize and examine. To provide similar information to a pathologist, it is therefore necessary to colourmap the PARS images in a similar manner. H&E is the most commonly used staining media in histology with the hematoxylin staining DNA as purple and eosin staining cytoplasm as pink. To emulate these colours, an inhouse developed software maps the pixel intensities of the 250 nm image to a purple colour to simulate staining with hematoxylin. Similarly, the 420 nm image is mapped to a pink hue to resemble staining with eosin. Once the individual images are colorized, their saturation levels are adjusted and a gaussian smoothing filter is applied to reduce colour noise. The colourized images are then converted to CYMK color space as it was found to be more consistent across different images. This is likely a result of the CMYK-space subtractive nature being a close analogue to the transmission loss of light through dye mixtures. The CMYK images are then added together and converted back to RGB color space for display purposes.

## Supplementary information

Supplementary Information
