# Supplementary material for: Reflection-mode virtual histology using photoacoustic remote sensing microscopy
Source: Sci Rep. 2020 Nov 5;10:19121. doi: 10.1038/s41598-020-76155-6 (PMC7644651; doi:10.1038/s41598-020-76155-6)
Supplement: Supplementary file 1 — Supplementary Information [file 41598_2020_76155_MOESM1_ESM.docx]

**Reflection-mode virtual histology using photoacoustic remote sensing microscopy**

**Kevan Bell^1,2,^**^†^**, Saad Abbasi^1,^**^†^**, Deepak Dinakaran^2,3^, Muba Taher^4^, Gilbert Bigras^5^, Frank K.H. van Landeghem^6^, John R. Mackey^3^, Parsin Haji Reza^1*^**

1. PhotoMedicine Labs, Department of Systems Design Engineering, University of Waterloo, Waterloo, Ontario, N2L 3G1, Canada
2. illumiSonics, Inc., Department of Systems Design Engineering, University of Waterloo, Waterloo, Ontario, N2L 3G1, Canada
3. Department of Oncology, University of Alberta, Edmonton, Alberta, T6G 1Z2, Canada
4. Division of Dermatology, Department of Medicine, University of Alberta, Edmonton, Alberta, T6G 2V1, Canada
5. Department of Laboratory Medicine and Pathology, University of Alberta, Edmonton, Alberta, T6G 2V1, Canada
6. Faculty of Medicine & Dentistry – Laboratory Medicine & Pathology Department, University of Alberta, Edmonton, Alberta, T6G 2B7, Canada

†. Equal contributions.

**Corresponding Author:* phajireza@uwaterloo.ca

**1. Ultraviolet DNA/Nuclear Contrast**

Previous investigations in photoacoustic imaging of cell nuclei have primarily used UV-C wavelengths typically around 266 nm [1] which aim to target the 260 nm absorption peak of DNA. However, 266 nm may not necessarily provide the greatest contrast against surrounding tissue regions. This disconnect between maximizing for absolute signal strength (i.e. targeting the absorption peak) and maximizing contrast may result from the non-zero UV contributions from neighboring chromophores such as other macromolecules in the nucleus and extranuclear structures of the cells, in the case of FFPE samples, the background paraffin. As such, we sought the optimal wavelength for nuclear contrast.

A 1 mg/mL solution of DNA (DNA from human placenta, Millipore Sigma) was prepared and placed directly onto the UV window of the tunable PARS. The excitation wavelength was then swept to produce a PARS-based optical absorption spectrum. This is shown in Figure SI1a. The overall shape is similar to that reported by more conventional spectroscopy techniques [2], with only a small shift in the locations of the peak and trough inside the UV range. It suggests that the 260 nm-range may be optimal for providing DNA signal strength, however, as will be shown in the next experiments this is not necessarily optimal for recovery of nuclear structure within tissues, which may be explained by the presence of other nuclear structures aside from DNA, such as histones, ribosomes and other specialized nuclear proteins [3].


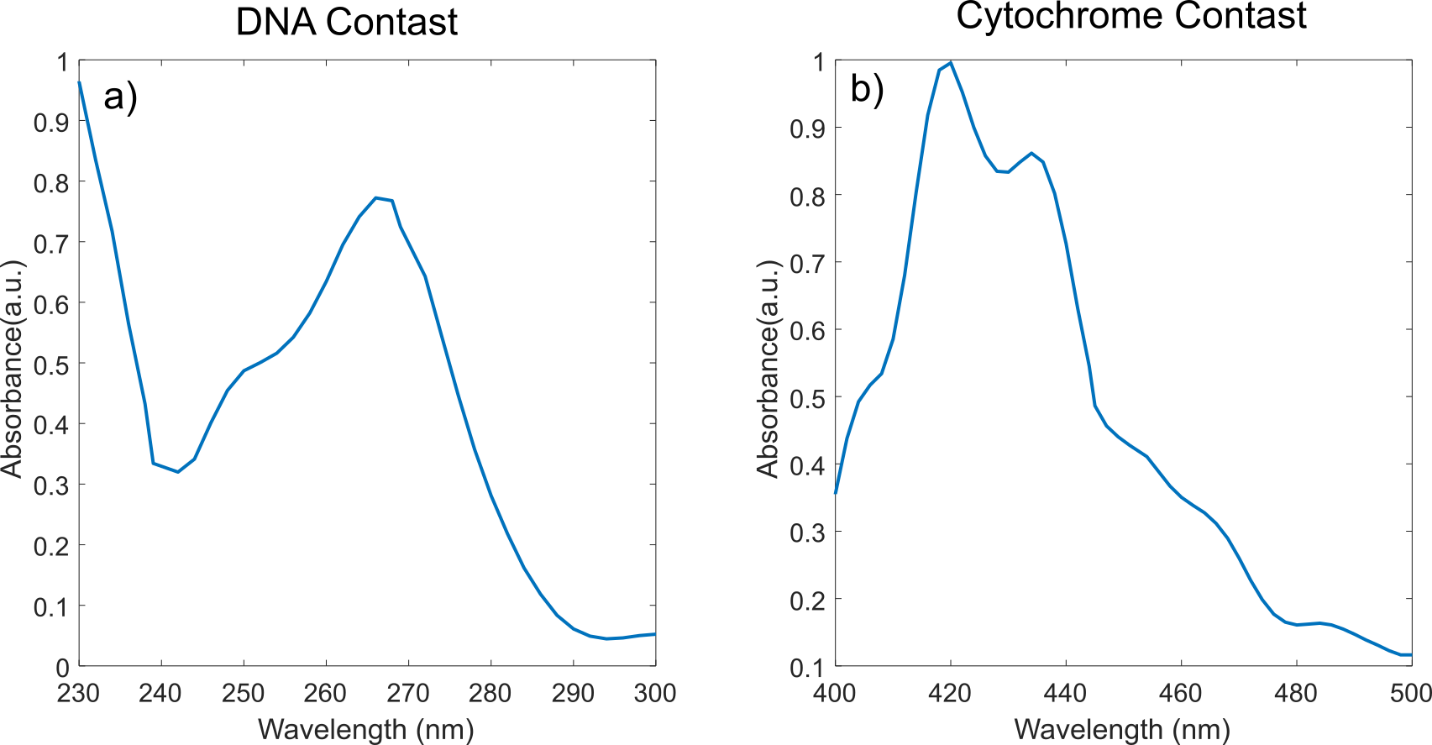


**Fig. SI1** Optical absorption spectra captured by the tunable PARS system for (a) DNA and (b) Cytochrome solutions.

A study was conducted to explore optimal excitation wavelengths for extracting the nuclear structure from FFPE samples. The study consisted of imaging the same region of a FFPE breast slide with multiple excitation wavelengths. An example of the results from this are shown in Figure SI2. Similar to previously reported studies on this topic looking at contact-based photoacoustic contrast, it was found that 250 nm appeared to give the best contrast for nuclei against the background. For this reason, the two-color PARS used 250 nm for DNA contrast.


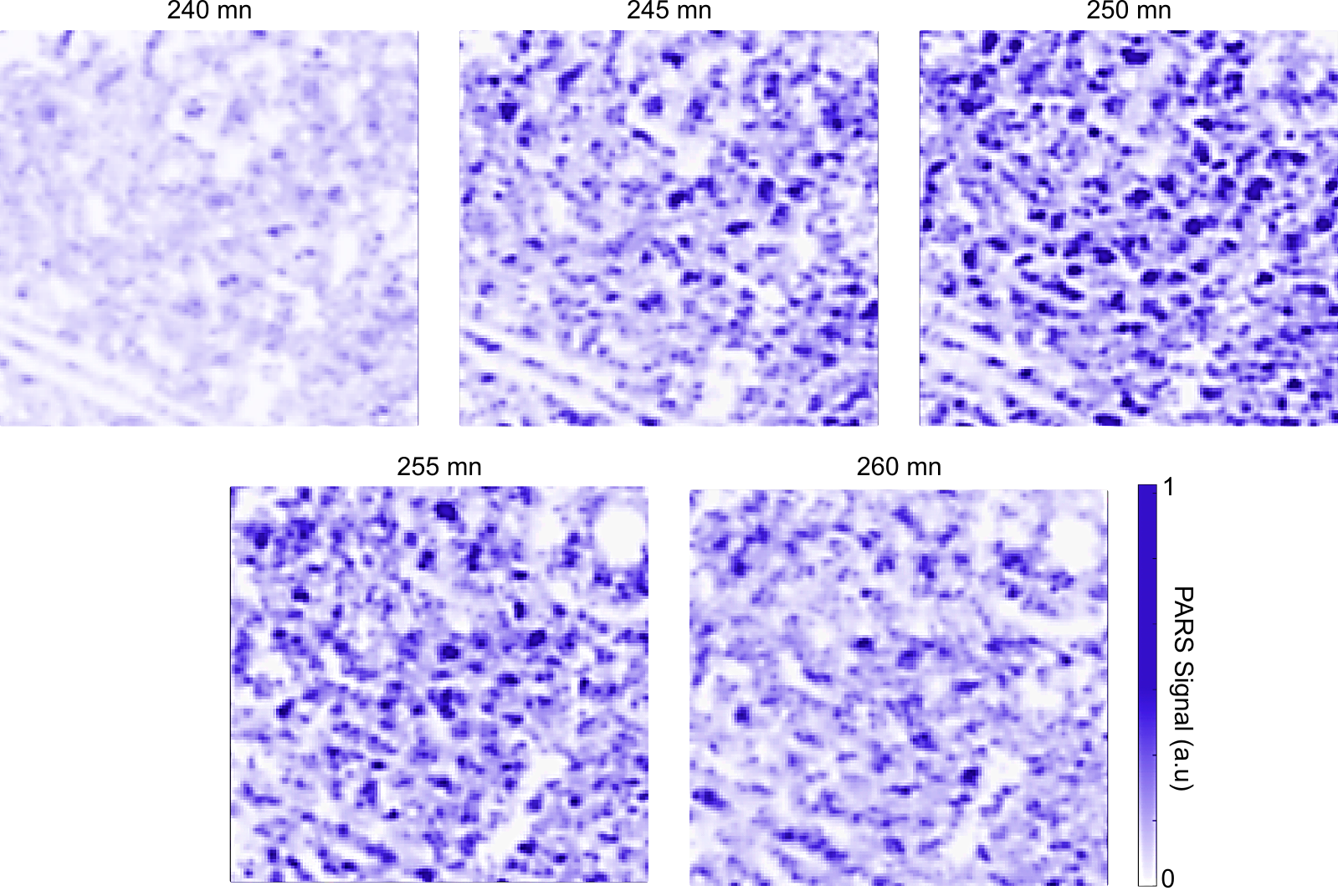


**Fig. SI2** A summary of a nuclear contrast study performed on the tunable PARS system.

**2. Blue/Green Hemeprotein Contrast**

Conventional H&E tissue preparations highlight both nuclear contrast along with that of the surroundings cytoplasm. With the UV excitation providing nuclear structure, cytoplasm may be recovered by targeting hemeproteins such as cytochromes. These hemeproteins are known to provide similar absorption spectra to that of hemoglobin; however, they appear in significantly lower concentrations within the surrounding tissue as compared to hemoglobin within erythrocytes. Cytochromes offer a strong absorption peak around 420 nm with a smaller peak in the mid 500 nm range [4]. PARS has previously recovered erythrocytes with excitation operating at 532 nm [5], but this wavelength was inappropriate for cytoplasm recovery as it required high excitation pulse energies. As with the nuclear contrast, we sought the optimal excitation wavelength for cytoplasm recovery looking at reduced cytochrome C samples.

A 10 mg/mL solution of cytochrome (Cytochrome C from bovine heart, Millipore Sigma) was prepared and placed directly onto the UV window of the tunable PARS. The excitation wavelength was then swept to produce a PARS-based optical absorption spectrum. This is shown in Figure SI1b. As with the DNA samples, the overall shape is again similar to that reported by more conventional spectroscopy techniques, showing a mid 400 nm absorption peak. Since this value appears to be appropriate for extracting cytoplasm morphology, it was used directly. An excitation wavelength of 420 nm was selected for the two-color PARS to extract cytoplasm structure.

**3. System Layout**

Included is a more detailed layout of the systems used in this study.


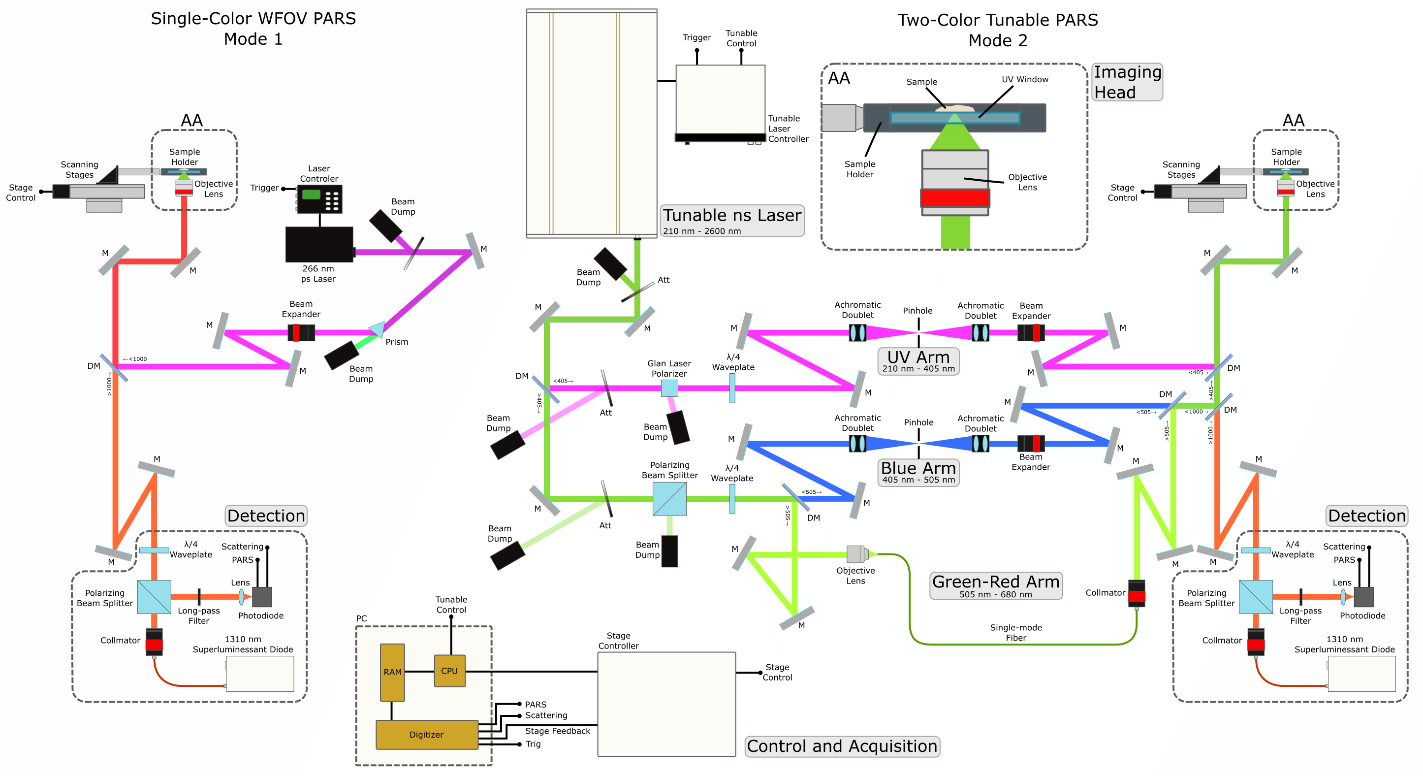


**Fig. SI3** An overview of the PARS systems used in the manuscript. The two-color tunable PARS is on the left. Excitation is provided by an Ekspla NT242 series nanosecond pulsed source. This beam is then split into three wavelength ranges (210 nm to 405 nm, 405 nm to 505 nm, and 505 nm to 680 nm) so that beam astigmatism and elipsisity may be improved through three spatial filters. These three paths are combined with the detection path, all of which are fed into the imaging head. The single-color PARS is shown on the right. It features a Bright Solutions picosecond pulsed source which emits primarily 266 nm but also contains some residual 532 nm. This 532 nm is removed using a dispersing prism. The excitation beam is then combined with the detection and fed into the imaging head. The control and acquisition architecture is highlighted in the bottom left. It features a digitizer which collects feedback information from the scanning stages, PARS signals, scattering signals, and trigger signals. This information is then streamed directly to the system RAM where it can be processed into images. Components are abbreviated as: Mirror (M), attenuator (Att.), and dichroic mirror (DM).

**4. Imaging Speeds and Field of Views**

The single-color acquisitions are produced using a 266 nm source operating at 50 kHz pulse repetition rate and a set of 2D linear-drive scanning stages. This apparatus can acquire images which are several centimeters in size with down to around 250 nm lateral sampling. Here, large images can be formed which may be useful for rapid gross assessment of samples with the presented architecture capable of forming 15 mm x 15 mm at a 4 µm lateral sampling in roughly 5 minutes. The latter a resolution of this system is fundamentally limited by optical diffraction, although more pragmatically limited by desired point density.

The tunable two-color architecture used to acquire full false-color H&E-like visualizations uses a much slower pulse repetition rate laser which can only operate up to 1 kHz. For this work, the system has been optimized for performance at the two characteristic wavelengths, 250 nm and 420 nm. Images are acquired using 2D screw-driven scanning stages. Although these are capable of wide scans, a reasonable limit is imposed due to the relatively slow interrogation rate of the system. For this reason, individual tunable frames are limited to 1.6 mm by 1.6 mm with 900 nm which are acquired in 54 minutes per wavelength. As with the stage scanning on the single-color modality, lateral resolution is primarily limited by lateral sampling of the stages.

**5. Sensitivity Study**

A study of the single-color (mode 1) and the two-color (mode 2) signal-to-noise ratio (SNR) performance is presented. Signal to noise (SNR) ratios are calculated from various data sets using the following definitions for SNR_max_ and SNR_mean_

$${SNR}_{max}=20log\left( \frac{Max\left( S_{i} \right)}{\sigma_{n}} \right)$$

$${SNR}_{mean}=20log\left( \frac{S_{i}}{\sigma_{n}} \right)$$

where $S_{i}$ is a collection of image pixels denoted as signal, and $\sigma_{n}$ is the standard deviation of a collection of image pixels denoted as noise. Values for the single-color and the two-color system for the four sample types are presented in Tables SI1 and SI2. As one example from this study, the max SNR on FFPE slides of human skin was measured to be roughly 55 dB using 20 nJ pulses in 266 nm on the single-color system. For another example, the max SNR on FFPE slides of human skin was measured to be roughly 41 dB using 35 nJ pulses in 250 nm and 26 dB using 120 nJ pulses in 420 nm on the two-color system.

**Table SI1**

| Single-Color PARS SNR | | | |
| --- | --- | --- | --- |
| **Sample Type** | **Wavelength (nm)** | **Max (dB)** | **Mean (dB)** |
| FFPE Slides | 266 | $55.0\pm3.6$ | $38.0\pm5.6$ |
| FFPE Blocks | 266 | $55.2\pm0.9$ | $34.7\pm2.6$ |
| Frozen Pathology | 266 | $53.7\pm12.5$ | $42.9\pm7.1$ |
| Fresh | 266 | $53.6\pm1.5$ | $38.4\pm2.5$ |

**Table SI2**

| Two-Color PARS SNR | | | |
| --- | --- | --- | --- |
| **Sample Type** | **Wavelength (nm)** | **Max (dB)** | **Mean (dB)** |
| FFPE Slides | 250 | $41.2\pm1.6$ | $25.9\pm1.4$ |
|  | 420 | $34.7\pm7.1$ | $16.3\pm1.7$ |
| FFPE Blocks | 250 | $46.2\pm4.3$ | $24.5\pm5.8$ |
|  | 420 | $31.1\pm2.7$ | $11.8\pm1.4$ |
| Frozen Pathology | 250 | $47.4\pm5.4$ | $36.4\pm4.4$ |
|  | 420 | $51.6\pm1.9$ | $32.3\pm22.7$ |
| Fresh | 250 | $44.6\pm5.9$ | $24.5\pm7.3$ |
|  | 420 | $46.5\pm4.2$ | $26.7\pm9.0$ |

# **References**

| [1] | T. T. Wong, R. Zhang, P. Hai, C. Zhang, M. A. Pleitez, R. L. Aft, D. V. Novack and L. V. Wang, "Fast label-free multilayered histology-like imaging of human breast cancer by photoacoustic microscopy," *Science advances,* vol. 3, no. 5, p. e1602168, 2017. |
| --- | --- |
| [2] | D.-K. Yao, K. I. Maslov, L. V. Wang, R. Chen and Q. Zhou, "Optimal ultraviolet wavelength for in vivo photoacoustic imaging of cell nuclei," *Journal of biomedical optics,* vol. 17, no. 5, p. 056004, 2012. |
| [3] | A. I. Lamond and W. C. Earnshaw, "Structure and function in the nucleus," *Science,* pp. 547-553, 1998. |
| [4] | S. Soltani, A. Ojaghi and F. E. Robles, "Deep UV dispersion and absorption spectroscopy of biomolecules," *Biomedical optics express,* vol. 10, no. 2, pp. 487 - 499, 2019. |
| [5] | S. Abbasi, M. Le, B. Sonier, K. Bell, D. Dinakaran, G. Bigras, J. R. Mackey and P. H. Reza, "Chromophore selective multi-wavelength photoacoustic remote sensing of unstained human tissues," *Biomedical Optics Express,* vol. 10, no. 11, pp. 5461 - 5469, 2019. |
